# Supplementary figures and images for: Functional analysis of African Xanthomonas oryzae pv. oryzae TALomes reveals a new susceptibility gene in bacterial leaf blight of rice
Source: PLoS Pathog. 2018 Jun 4;14(6):e1007092. doi: 10.1371/journal.ppat.1007092 (PMC6037387; doi:10.1371/journal.ppat.1007092)

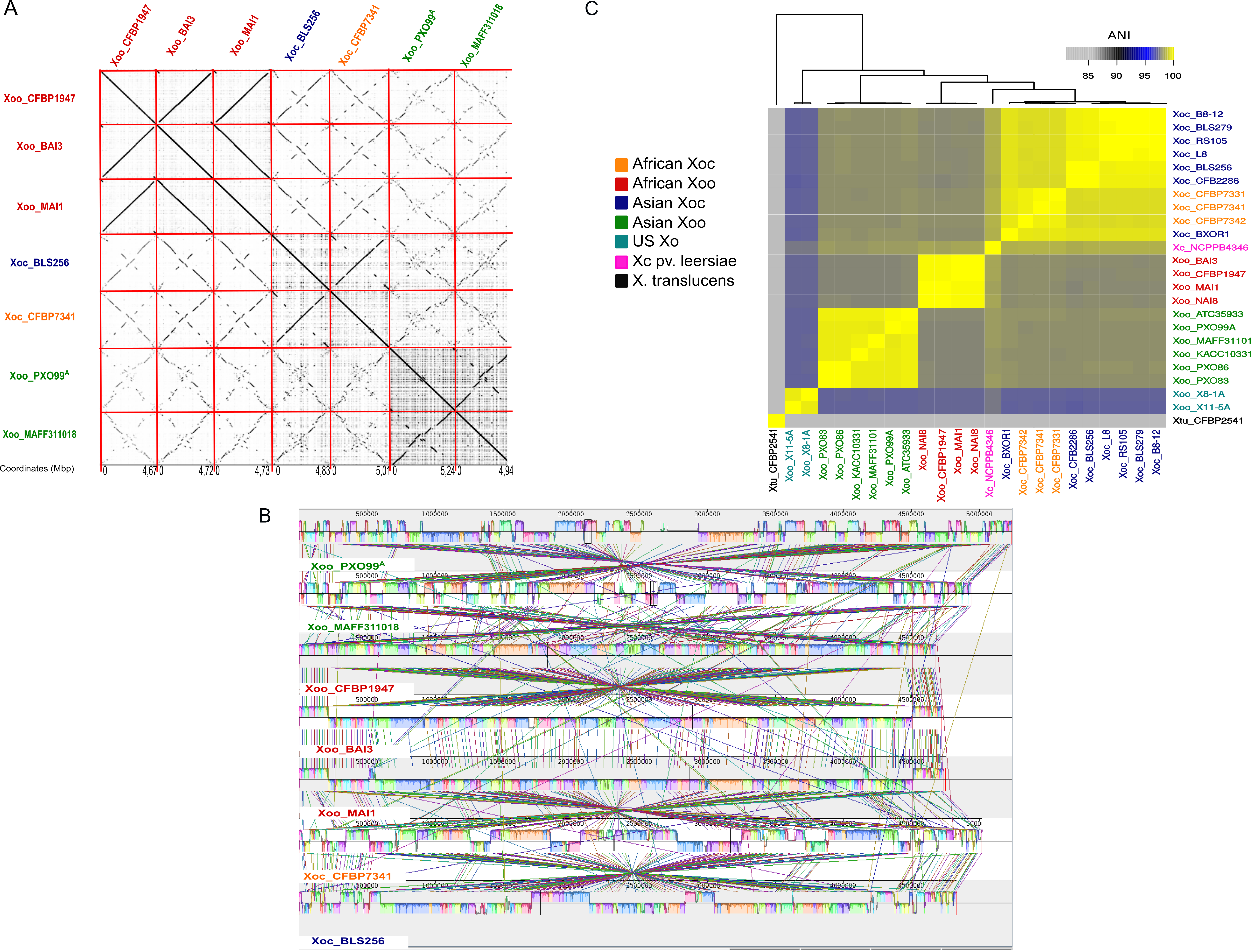

Supplement: S1 Fig — (A) Paired dot plots showing whole genome alignments between Xo genomes. Dotplots were made using Gepard [70] with word-size = 30 and window = 30. For visualization, the upper color limit was set to 50%, and the lower color limit and grayscale to zero. (B) Mauve multiple alignment of selected Xo genomes. (C) Heatmap shows average nucleotide identity (ANI) values for all pairs of genomes calculated using the ANI tool from the enveomics suite [44], top shows hierarchical clustering based on these values. (TIF) [file ppat.1007092.s001.tif]

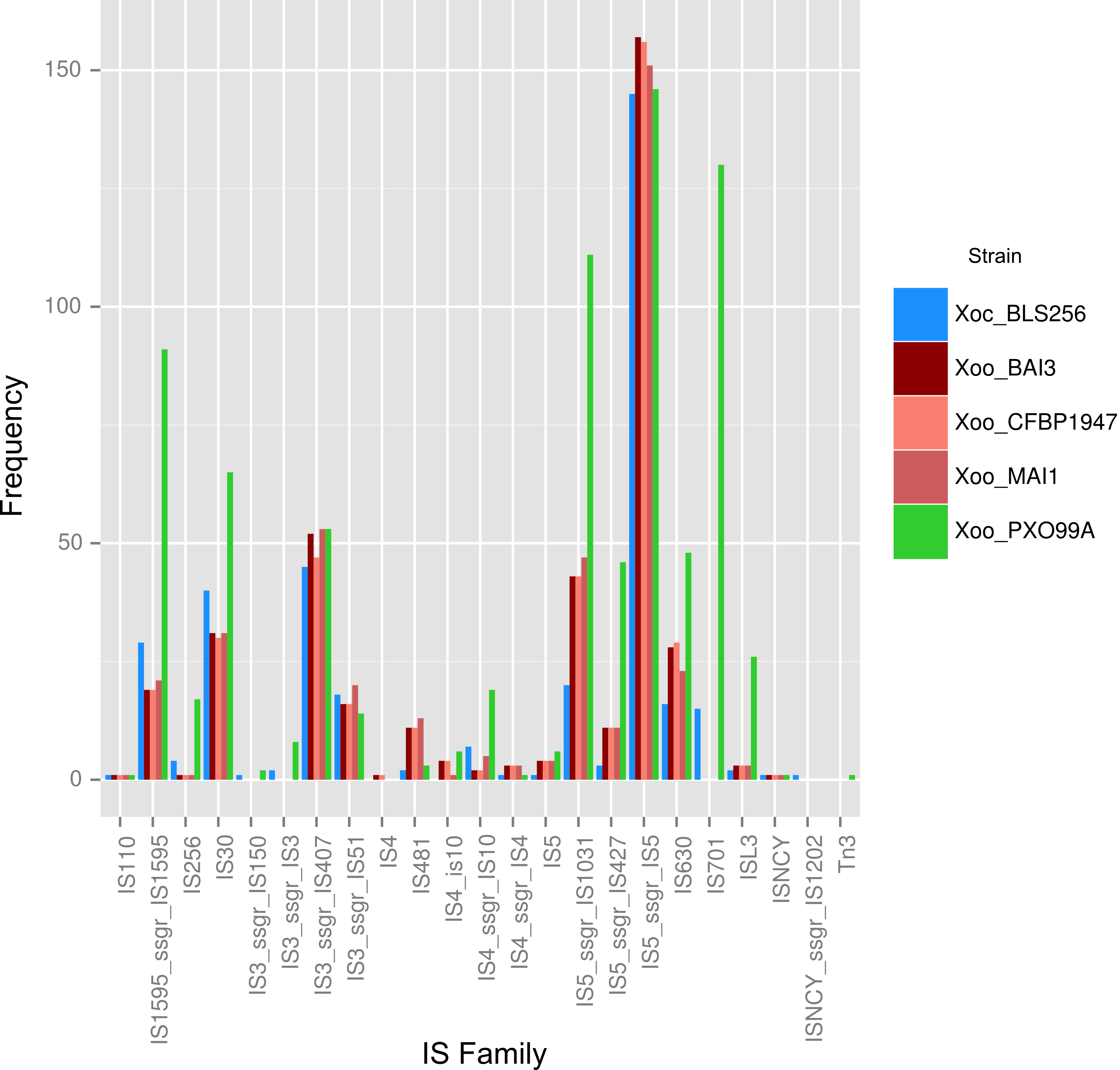

Supplement: S2 Fig — Frequency (total instances) of insertion elements identified in the genomes of African (MAI1, BAI3 and CFBP1947) and Asian (PXO99A) Xoo strains, and in Xoc strain BLS256. IS were identified using IS-Saga, and family names used in the IS-Saga database are shown [74]. TnXax1-like sequences were identified using BLAST. (TIF) [file ppat.1007092.s002.tif]

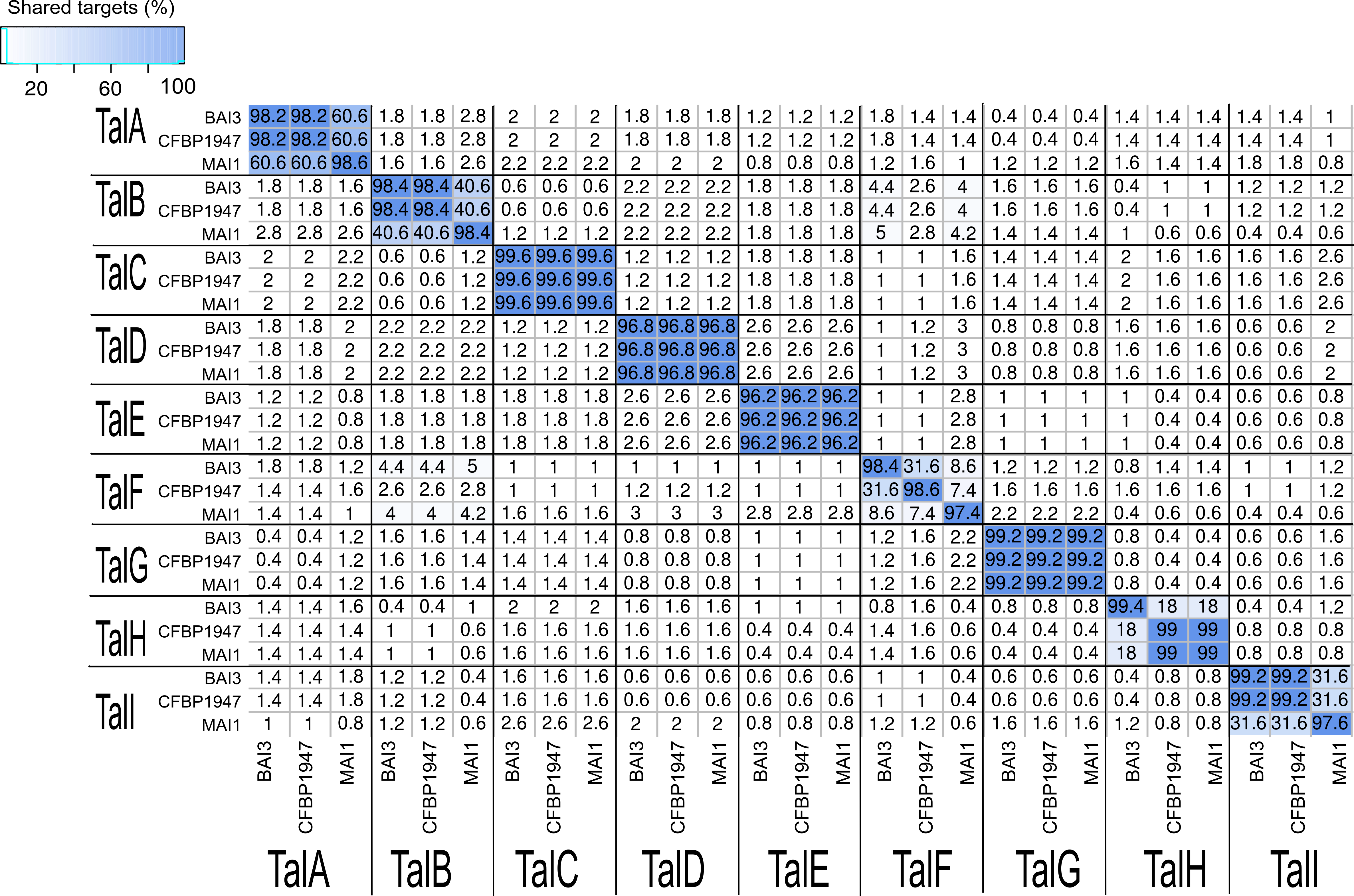

Supplement: S3 Fig — Heatmap showing the percentage of shared predicted targets for all pairs of African Xoo TAL effectors. Predictions were made using TALVEZ v.3.1 [62], and the top 500 predicted targets based on locus IDs were used for each comparison. (TIF) [file ppat.1007092.s003.tif]

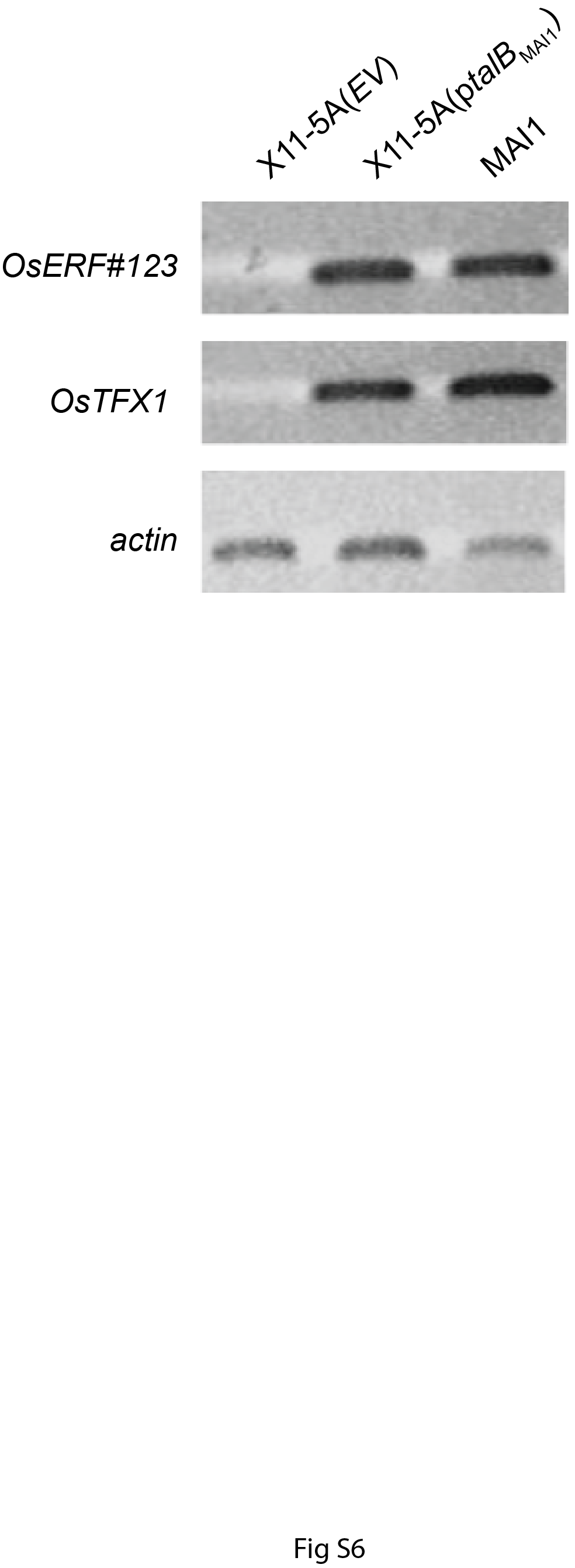

Supplement: S4 Fig — sqRT-PCR analysis of Os09g39810 (aka OsERF#123), Os09g29820 (aka OsTFX1) and actin expression levels 24 hours post infiltration of Azucena rice leaves with Xoo strain MAI1, and Xo X11-5A strains carrying talBMAI1 or an empty vector (EV). RNA quality and quantity was estimated using a ND-1000 Nanodrop spectrophotometer. cDNA was synthesized with 1μg of total RNA by means of the SuperScript first-strand synthesis system (Invitrogen, Carlsbad, CA, U.S.A.). (TIF) [file ppat.1007092.s004.tif]

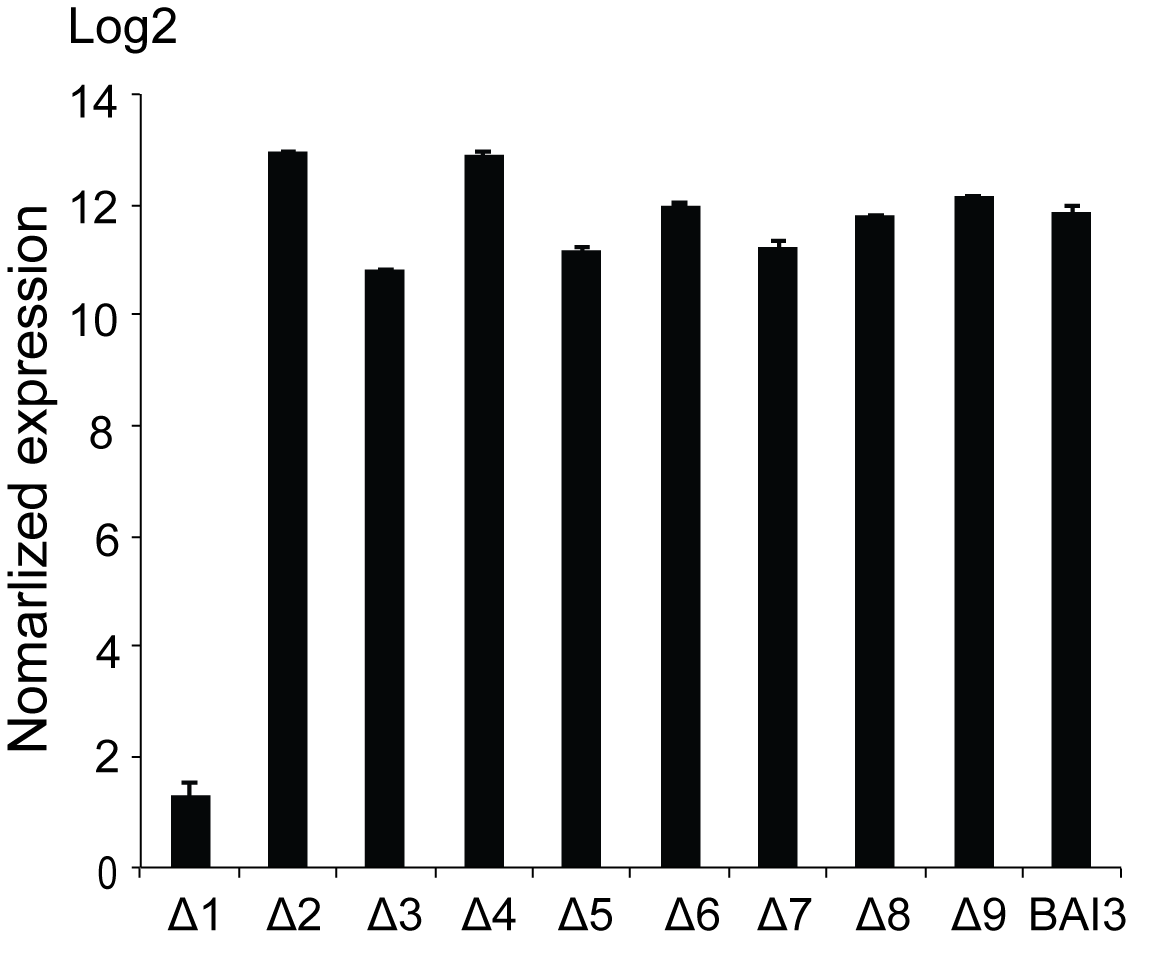

Supplement: S6 Fig — In order to identify a talB mutant, a library of BAI3 TAL effector gene mutants (Δ1 to Δ9) was screened for its ability to induce OsERF#123. The expression level of OsERF#123 was evaluated by quantitative reverse transcription polymerase chain reaction (qRT-PCR) 24 hours after syringe infiltration of Nipponbare rice leaves. The wild type strain BAI3 was used as control. Gene expression was normalized against water inoculated leaves. Error bars represent +/- SD based on three technical replicates. (TIF) [file ppat.1007092.s006.tif]
