# Supplementary material for: Functional analysis of African Xanthomonas oryzae pv. oryzae TALomes reveals a new susceptibility gene in bacterial leaf blight of rice
Source: PLoS Pathog. 2018 Jun 4;14(6):e1007092. doi: 10.1371/journal.ppat.1007092 (PMC6037387; doi:10.1371/journal.ppat.1007092)
Supplement: S5 Fig — RNA collected 24 hours post-infiltration of rice leaves with Xoo strains MAI1 and X11-5A (ptalB) were subjected to 5’ RACE. Obtained cDNA sequences were aligned above (MAI1) and below (X11-5A (ptalB)) the predicted 5’-UTR of OsERF#123. The effector binding element (EBE) for TalB is highlighted in gray. Numbering starts downstream of the 3’ end of the EBE. The total number of sequences obtained (N) is given on the left. (DOCX) [file ppat.1007092.s005.docx]

**(4) ATTCTCCCGCCGAGCGGAGC…**

**(2) TCATTCTCCCGCCGAGCGGAGC…**

**(10) ATTCATTCTCCCGCCGAGCGGAGC…**

**(3) AACCCCCGCCCCCGCCGACTCCCATTCATTCTCCCGCCGAGCGGAGC…**

**1........10........20.........30........40........50........60........70........80........**

**T**GCGATGCGTTTCCCACCTCCCACCTCCCGGCTCTGCTTCTGTCTCTCTCCCTCTGCTTCCTTTTAAACAACCCCCGCCCCCGCCGACTCCCATTCATTCTCCCGCCGAGCGGAGC…

**(6) AACCCCCGCCCCCGCCGACTCCCATTCATTCTCCCGCCGAGCGGAGC…**

**(10) ATTCATTCTCCCGCCGAGCGGAGC…**

**(4) ATTCTCCCGCCGAGCGGAGC…**

**Fig. S5 *OsERF#123* transcription start sites observed in response to TalB**

RNA collected 24 hours post-infiltration of rice leaves with *Xoo* strains MAI1and X11-5A (p*talB*) were subjected to 5’ RACE. Obtained cDNA sequences were aligned above (MAI1) and below (X11-5A (p*talB*)) to the predicted 5’-UTR of *OsERF#123*. The effector binding elements (EBE) for TalB is highlighted in gray. Numbering starts downstream of the 3’ end of the TalB EBE. The total number of sequences obtained (N) is given on the left.
