# Supplementary material for: Functional analysis of African Xanthomonas oryzae pv. oryzae TALomes reveals a new susceptibility gene in bacterial leaf blight of rice
Source: PLoS Pathog. 2018 Jun 4;14(6):e1007092. doi: 10.1371/journal.ppat.1007092 (PMC6037387; doi:10.1371/journal.ppat.1007092)
Supplement: S4 Table — (DOCX) [file ppat.1007092.s010.docx]

**Table S4.** Primer sequences used in this study.

| **Name** | **Sequence (5’->3’)** | **Application** |
| --- | --- | --- |
| OsERF#123-Fw | ACATCTACGCCCGTCAACTA | QRT-PCR |
| OsERF#123-Rev | TGGTGTGGTCTCGGAGCATC | QRT-PCR |
| OsERF#123SQ-Fw | GCGCGGCGCCAACGCCGTCC | sQRT-PCR |
| OsERF#123SQ-Rev | CTTGTTGGTGGTGTGGTCTC | sQRT-PCR |
| OsTFX1-Fw | ACTGCCTCTCACCTCCAAGC | QRT-PCR & sQRT-PCR |
| OsTFX1-Rev | GGTAGGCGTCATCTGTGCTG | QRT-PCR & sQRT-PCR |
| Actin-Fw | TGTGTGTGACAATGGAACTGGC | QRT-PCR |
| Actin-Rev | GAGTCCAACACGATACCAGTTG | QRT-PCR |
| ActinSQ-Fw | GTTACGTCGCGGCTGATTATG | sQRT-PCR |
| ActinSQ-Rev | TCGTGTTGGAGCATTTCCTTC | sQRT-PCR |
| OsERF#123-Rev2 | CTTGTTGGTGGTGTGGTCTC | 5’-RACE |
