# Supplementary material for: Functional analysis of African Xanthomonas oryzae pv. oryzae TALomes reveals a new susceptibility gene in bacterial leaf blight of rice
Source: PLoS Pathog. 2018 Jun 4;14(6):e1007092. doi: 10.1371/journal.ppat.1007092 (PMC6037387; doi:10.1371/journal.ppat.1007092)
Supplement: S5 Table — (DOCX) [file ppat.1007092.s011.docx]

**Table S5.** RVD sequences of the dTALEs used in this study and their target sequence.

| **Gene name** | **dTALE** | **RVD sequence^a^** | **Target site^b^** |
| --- | --- | --- | --- |
| *OsERF#123* | dTALE_ERF-1_ | NN-NG-NI-HD-NI-NI-HD-NI-HD-NI-NG-NI-NI-NI-NN-NG-NG-NG | **T**GTACAACACATAAAGTTT |
|  | dTALE_ERF-2_ | HD-HD-NI-HD-NI-NI-NI-NG-NN-HD-NN-NI-NG-NN-HD-NN-NG-NG | **T**CCACAAATGCGATGCGTT |
| *OsTFX1* | dTALE_TFX-1_ | NN-NG-NI-HD-HD-NI-NI-NI-NG-HD-NN-NI-HD-NI-NI-NI-NI-NG | **T**GTACCAAATCGACAAAAT |
|  | dTALE_TFX-2_ | NI-NN-NN-HD-NI-NI-NG-HD-NI-NI-NI-NI-NN-HD-NI-HD-HD-NG | **T**AGGCAATCAAAAGCACCT |

^a^Amino acids in one letter code.

^b^The 5′ terminal nucleotide of each target box is indicated in bold.
